# N‐of‐1 trials in clinical research: Methodological foundations, statistical approaches and implementation challenges

**DOI:** 10.1002/bcp.70382

**Published:** 2025-12-08

**Authors:** Marcos Clint Leal De Carvalho, Matheus de Matos Dourado Simões, Ubiratan Cardinalli Adler, Antonio Brazil Viana Junior, Caren Nádia Soares de Sousa, Lia Lira Olivier Sanders

**Affiliations:** ^1^ Postgraduate Program in Translational Medicine, School of Medicine Federal University of Ceará Fortaleza CE Brazil; ^2^ Walter Cantídio University Hospital, Medical Residency Program Federal University of Ceará Fortaleza CE Brazil; ^3^ Department of Medicine Federal University of São Carlos São Carlos SP Brazil; ^4^ Research Management Unit – Brazilian Hospital Services Company (EBSERH) Fortaleza CE Brazil; ^5^ Neuropsychopharmacology Laboratory, Drug Research and Development Center, Department of Physiology and Pharmacology, School of Medicine Federal University of Ceará Fortaleza CE Brazil; ^6^ School of Medicine, Department of Clinical Medicine Federal University of Ceará Fortaleza CE Brazil

**Keywords:** data analysis, N‐of‐1, personalized medicine

## Abstract

N‐of‐1 trials offer a unique and rigorous methodology for evaluating individualized treatment responses, particularly within the context of personalized medicine. This article provides a comprehensive explanation of the conceptual and methodological underpinnings of N‐of‐1 trials, with particular emphasis on statistical techniques and considerations critical for their design and analysis. Existing guidelines for planning and conducting these studies are summarized, along with a discussion of the practical and theoretical challenges to their implementation in clinical practice. We provide an overview for clinicians and researchers who may be unfamiliar with the design. As most of the existing guidance has focused on design and implementation considerations, we expand on the statistical analysis. We aim to support researchers and methodologists in understanding and advancing the methodological toolkit necessary for high‐quality N‐of‐1 research.

**What is already known about this subject**

- N‐of‐1 trials provide a framework to evaluate treatment efficacy in a single patient.
- They are relevant tools for personalized medicine but remain underused in clinical research.
- Barriers include design complexity, statistical challenges and limited clinician awareness.

**What this study adds**

- Integrates methodological and statistical foundations underlying N‐of‐1 trial design.
- Summarizes a broad range of statistical approaches, from classical tests to modern time‐series and Bayesian methods.
- Highlights key challenges and practical strategies for wider clinical implementation.

## 1 INTRODUCTION

N‐of‐1 studies have become an innovative approach for evaluating treatments and health interventions in contemporary clinical research.^1^, ^2^ Unlike traditional clinical trials, which analyse large groups of patients, N‐of‐1 studies focus on a single individual, allowing for detailed and individualized investigations of responses to different interventions. This methodology is particularly advantageous in areas such as personalized medicine, where individual variations can significantly influence the effectiveness of treatments.^3^

This type of study plays a crucial role in the new medical paradigm, allowing clinicians to tailor treatment plans to each patient's needs. They can also produce valuable and meaningful evidence for general medical practice, especially when randomized clinical trials are not feasible.^4^

By focusing on truly effective interventions for patients, N‐of‐1 studies can reduce the costs associated with ineffective treatments. Patients who receive more appropriate therapies are less likely to experience unwanted side effects or complications, which reduces the cost of hospitalizations and new medications.^5^ Additionally, patients who respond well to a specific therapy may require less follow‐up care, thereby freeing up resources and time for both the patient and the healthcare provider. This optimizes the allocation of clinician time and improves the patient experience.^6^

N‐of‐1 studies are already being utilized in fields such as education, psychology and the broader research literature on behaviour change. In these studies, researchers investigated how an individual responds to various interventions.^6^, ^7^, ^8^, ^9^, ^10^ Wide‐ranging applications across different disciplines can make it challenging for researchers to grasp the essential concepts and methods involved in N‐of‐1 trials.^11^ Furthermore, researchers in the medical field tend to be more familiar with traditional randomized controlled trials. Therefore, we aim to equip clinicians and researchers with the necessary knowledge to plan and execute N‐of‐1 trials, including their applications, methodologies and statistical analyses.

In this context, N‐of‐1 studies provide a unique opportunity to explore variability in patient responses and optimize interventions based on individual‐specific data. By integrating rigorous data collection and analysis methodologies, these studies can enhance the understanding of health conditions and improve clinical decision‐making. This article aims to discuss the principles, applications and challenges of N‐of‐1 studies, highlighting their transformative potential in medical practice and health research. We provide an overview for clinicians and researchers who may be unfamiliar with the design. As most of the existing guidance has focused on design and implementation considerations, we expand on the statistical analysis.

### 1.1 Scientific and clinical value of N‐of‐1 trials

Often called personalized trials, N‐of‐1 trials are a specific form of single‐case experimental design (SCED). SCEDs are a subset of experimental designs comprising case descriptions, nonrandomized and randomized designs.^12^ They are used to study the relationship between one or more treatments or levels of treatment and changes in biological or behavioural outcomes. These designs first appeared in initial experimental psychology research and were subsequently developed and systematized in basic and applied behaviour analysis studies and were later expanded to fields such as medicine, public health, education, counselling psychology, clinical psychology, health behaviour and neuroscience.^13^

**FIGURE 1:**
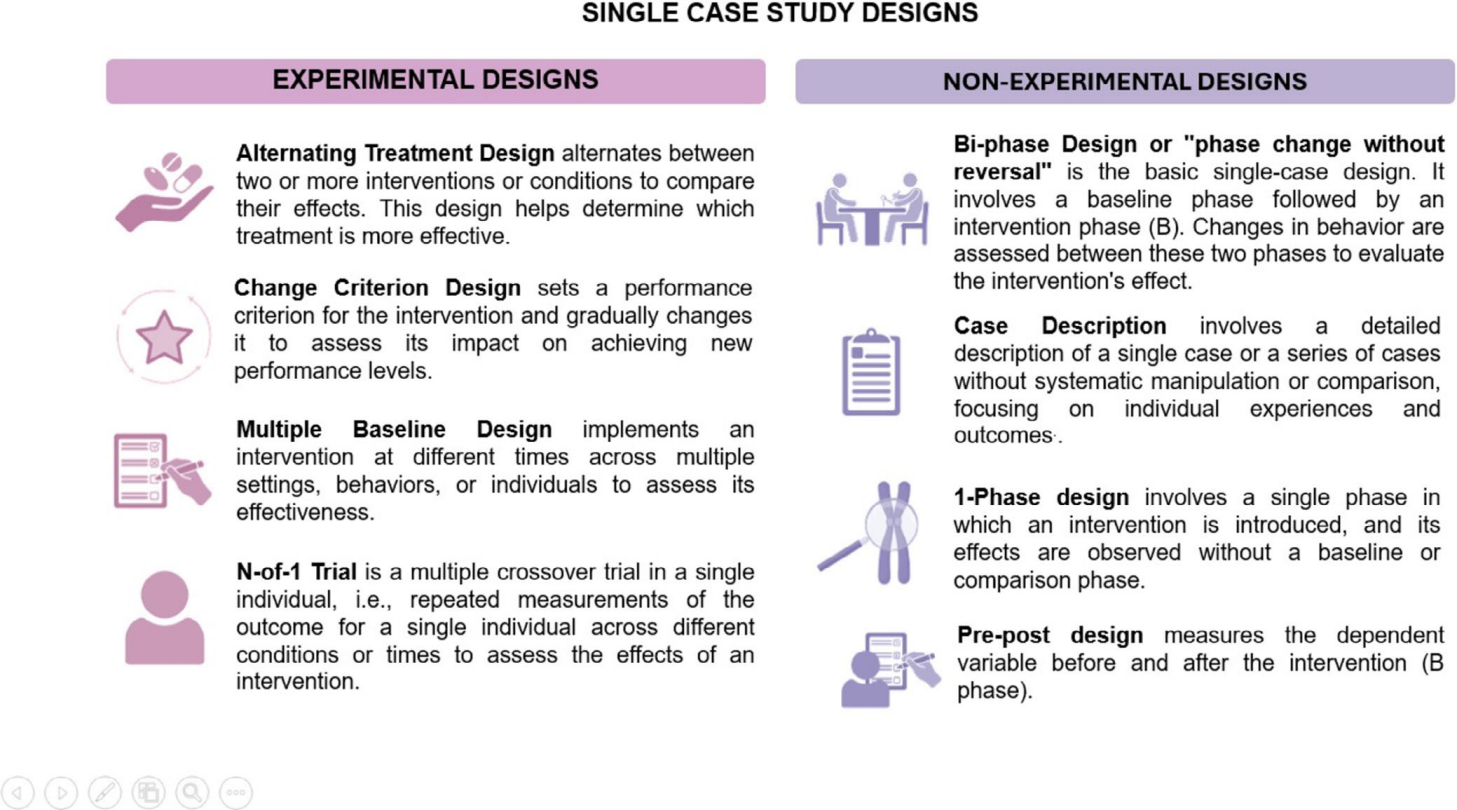
Standard single‐case study designs.

SCED studies repeatedly measure a dependent variable in the absence and presence of an independent variable (e.g., applying an intervention). Each participant serves as their control by allowing the measurement of the dependent variable at baseline (i.e., before the intervention) and during or after the intervention. Numerous specific experimental and non‐experimental study designs are derived from this framework (Figure 1). The main types of SCED are ABAB, multiple baseline and alternating treatment designs.^14^ In the medical field, the most commonly used design is the ABAB design, which includes the N‐of‐1 trial, which is the focus of this paper. Importantly, in fields such as the behavioural and social sciences, the term “N‐of‐1 trial” is applied to various forms of SCEDs; however, in the medical field, the term has a more restrictive use, as discussed below.

In 1953, Hogben and Sim presented N‐of‐1 trials to physicians. However, it took another thirty years for the movement to find an effective spokesperson, Gordon Guyatt of McMaster University. Since then, over 2000 patients have participated in published N‐of‐1 trials.^15^ In the current medical literature, the term “N‐of‐1 trial” is used to describe a prospectively planned, multiple crossover trial in a single individual—often challenge‐withdrawal‐challenge‐withdrawal (the SECD “ABAB” design), in which one period (“A”) is the treatment studied and the other period (“B”) is a comparison treatment, a control, or no intervention.^16^ Researchers and practitioners in healthcare are increasingly adopting the N‐of‐1 trial design as an alternative to randomized controlled trials (RCTs)^17^ and as a source of customized data to improve patient care immediately.^18^ We address these topics in the following sections.

### 1.2 N‐of‐1 trials as alternatives to randomized controlled trials (RCTs)

Although RCTs remain the gold standard for determining treatment effectiveness, they have several limitations. For example, RCTs provide only an idea of the average effect of an intervention on outcomes. Variability in how people react to the intervention is inevitable, leading to heterogeneity in treatment effects (HTEs). Research on HTE has shown variability in outcomes across RCTs. In some studies, very few people exhibited the benefits of that treatment.^19^ The limited external validity and generalizability of RCTs are also relevant issues. Patients with comorbid conditions and concurrent treatments are often excluded from RCTs, even though these patients are most common in clinical practice. Restrictive inclusion and exclusion criteria might only allow fewer than 10% of people with the disease under consideration to enrol in RCTs.^20^

N‐of‐1 trials usually adopt more open inclusion criteria and document comorbidities rather than excluding them. They allow patients that physicians treat in everyday clinical practice to participate in the study, providing real‐world data. Thus, it has been suggested that future trials should be grounded in N‐of‐1 and aggregated N‐of‐1 designs, emphasizing simultaneously investigating multiple phenotypes and uncovering causal relationships between them.^21^

Furthermore, N‐of‐1 trials can minimize significant costs compared with RCTs. A recent study has shown that N‐of‐1 trials can test interventions with adequate power with far fewer patients than traditional RCTs and crossover designs.^17^ In addition to reducing costs, the potential to study a limited number of patients is crucial in investigating rare diseases.^22^

### 1.3 N‐of‐1 trials and personalized medicine

The discovery of considerable variation in illness manifestations and treatment responses among individuals with illnesses led to the development of personalized medicine. N‐of‐1 trials provide a framework for determining the most effective treatment for individual patients. They offer deep insights into human biology by enabling personalized causal assessments of how interventions impact individual health. It is possible to evaluate whether interventions work and, more importantly, for whom. Unlike larger studies where results take time to benefit participants, N‐of‐1 trials yield immediate, personalized health insights. Additionally, by aggregating results across individuals, these trials can uncover broader patterns of response, helping to identify which interventions are most effective in specific populations.^21^ This customized approach can enhance treatment efficacy, minimize adverse effects and improve patient adherence and satisfaction.^23^ Additionally, by allowing for the direct estimation of individual treatment effects, N‐of‐1 trials can facilitate more precise and personalized care, potentially improving patient outcomes and reducing healthcare costs.^24^

N‐of‐1 trials are most suitable in scenarios of clinical equipoise, where uncertainty persists regarding the comparative efficacy of different treatment options. In addition to this more prominent use of evaluating pharmacological treatment effectiveness, N‐of‐1 trials apply to various situations, such as assessing treatment harm, nonpharmacological interventions, lifestyle changes and rare diseases.^18^

### 1.4 Other indications

N‐of‐1 trials hold promise in assessing additional indications for drugs or interventions, known as treatment repositioning.^21^ If there is a belief that a drug originally intended for one clinical profile or condition could benefit patients with different profiles or conditions, conducting individualized trials comparing the drug to standard treatments tailored to patient characteristics is warranted. Positive findings from these nuanced trials may warrant further investigation in larger, conventional trials for broader applications.

N‐of‐1 trials may also help guide deprescribing decisions, especially when the benefits of continued treatment are unclear. In such cases, clinicians and patients are often reluctant to stop medications. By focusing on the individual's response, comparing the effects of continuation with a current treatment *vs*. no treatment or placebo, these trials can reduce uncertainty and support more confident, shared decisions. Goyal and colleagues recently demonstrated this approach by comparing ongoing beta‐blocker use with deprescribing in patients with heart failure and preserved ejection fraction.^25^

Well‐designed N‐of‐1 trials can also play a role in early‐phase research by assessing the tolerability, dosing and potential utility of experimental compounds.^5^ They use objective, data‐driven criteria to determine the optimal or best intervention for a patient. For example, they have been proposed as a “preproof‐of‐concept” approach in chronic pain management, where they can rapidly determine the efficacy of a therapeutic candidate by comparing drug effects with placebo or standard care.^26^ Additionally, in cancer drug development, N‐of‐1 trials are emerging as powerful tools to accelerate drug evaluation in the precision oncology era, enabling a more personalized and patient‐focused approach.^82^

Finally, N‐of‐1 trials provide valuable opportunities for physician training by exposing them to objective clinical decision‐making and evidence‐based practice at a systematic and rigorous level. They enhance physicians' sensitivity to individual patient nuances while demanding familiarity with trial design, execution and ethical considerations related to patient involvement in research.^5^

## 2 N‐OF‐1 METHODOLOGY/GUIDELINES

**FIGURE 2:**
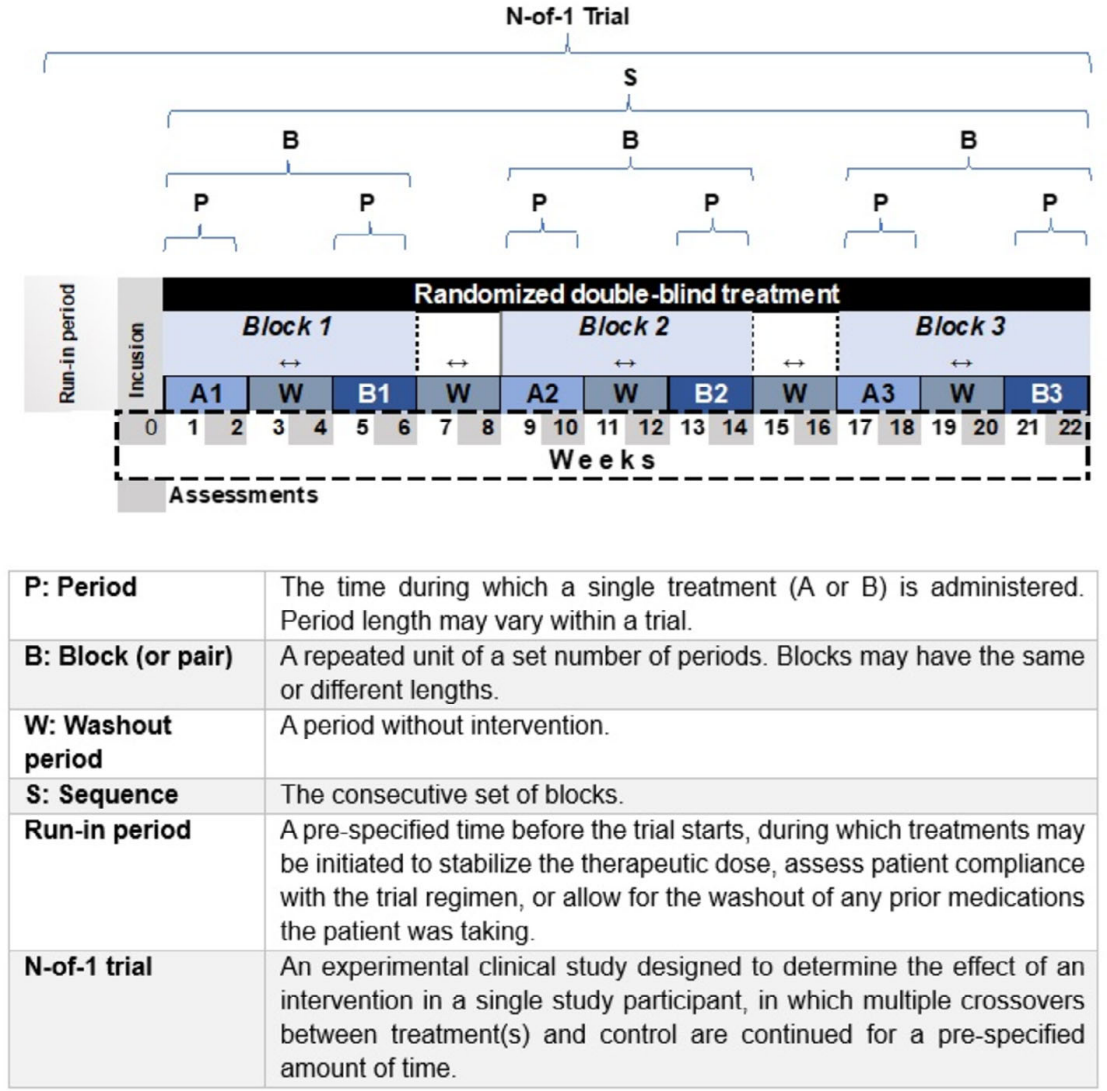
Methodological terminology for N‐of‐1 trial reports.

There are two main guidelines for N‐of‐1 trials. The *SPIRIT extension for n‐of‐1 trials reporting guideline and checklist* (SPENT)^28^ guides the development and publishing of n‐of‐1 trial protocols. The *CONSORT Extension for Reporting N‐of‐1 Trials (CENT)* ^16^, ^29^ offers instructions for reporting individual and series of N‐of‐1 trials. Both guidelines are aligned with each other, using similar wording whenever possible. This alignment ensures a seamless progression from protocol development to the final trial report, thereby facilitating the researcher's report production and enabling an assessment of its adherence to the protocol. Considering the possibility of confusion regarding the varying use of the terms N‐of‐1 trial and SCED, clearly defining the terminology used in the medical field for N‐of‐trials is essential. Figure 2 illustrates the methodological terminology CENT guidelines recommend for N‐of‐1 trial reports, showing a typical N‐of‐1 trial design.

The CENT and SPENT guidelines are comprehensive, in‐depth documents that deserve careful reading. Nevertheless, researchers and clinicians embarking on their exploration of this area may need a starting roadmap to navigate the critical stages of N‐of‐1 trial design, implementation and analysis, with a focus on fundamental principles and practical considerations. Guyatt et al.’s guideline^30^ for performing clinical N‐of‐1 trials can serve as a starting point. We integrated their crucial advice with the main CENT and SPENT recommendations in 10 steps, which summarize the key recommendations provided by the available guidelines.

### 2.1 Key steps for planning and conducting N‐of‐1 trial

Step 1 – assess the need for an N‐of‐1 trial
  Is the N‐of‐1 study the most suitable approach to address the specific clinical uncertainty at hand? This involves assessing the need for an individualized trial, clarifying which aspects of treatment effectiveness are unclear, and defining both primary and secondary outcome measures. Consider long‐term implications of treatment effects along with the possibility of conducting a series of N‐of‐1 trials to enhance generalizability and cumulative knowledge.
Step 2 – design the trial (use SPENT)
  After establishing the N‐of‐1 trial as the study design, the next consideration is the selection of study participants. In individual trials, eligibility criteria may not be applicable; researchers may describe patient characteristics, such as diagnosis, comorbid conditions and concurrent medications.^29^ For a series of N‐of‐1 trials, the inclusion and exclusion criteria for participants should be described in a manner similar to those in a regular randomized clinical trial.
  Trial design should adhere to the SPENT framework,^28^ addressing key domains, including feasibility, patient engagement, treatment characteristics and trial duration. Whether the study consists of a single N‐of‐1 trial or a series of trials should be clearly stated, as well as whether the design will be tailored to each participant. Researchers must also specify the study framework (superiority, equivalence, non‐inferiority, or exploratory).^28^
  The design should include definitions of outcome measures, criteria for stopping the study and the use of a run‐in period, if necessary. The logistical feasibility of implementation in clinical settings, the availability of support and resources and the selection of appropriate data analysis strategies should be considered. Ethical dimensions must be integrated early in the design to ensure responsible research conduct.
  Once the general design and participants are determined, it is necessary to describe and justify the choice of the comparators. N‐of‐1 trials mitigate concerns related to the placebo effect, as participants receive all comparator interventions.^28^ These concerns diminish when the N‐of‐1 trial assesses an add‐on intervention.^31^
  How and when interventions will be administered, including the planned number of periods, crossovers and duration of each period (run‐in and washout details, if applicable)^28^? In the case of a series of N‐of‐1 trials, researchers must detail how they will customize design components for individual participants.
  It is also crucial to define randomization procedures, including the method of assignment (e.g., sealed envelopes, centralized or computerized systems).
  The sample size is a different concept in N‐of‐1 trials. In an individual trial, sample size refers to the number of periods or measurements within a treatment period. Instead, in a series of N‐of‐1 trials, the sample size refers to the number of individual trials. For statistical analysis, the number of repeated periods across trials and repeated sampling within periods may be more important than the total number of trials.^29^ Research protocols should describe how these numbers can achieve study objectives.^28^ Plan statistical analyses in advance.
Step 3 – submit the protocol to a research ethics review committee (ERC)
  Prepare a detailed protocol and submit it for review by a research ethics committee. The submission addressed all relevant ethical concerns, and feedback from the committee was incorporated as needed to ensure the protocol's rigour and compliance with ethical standards.
Step 4 – register the study protocol in a clinical trials registry platform
  Prior to recruitment, register the finalized protocol in an appropriate clinical trials registry. This step ensures transparency and allows for public access to the trial design, with all necessary methodological details accurately reported in the registry.
Step 5 – recruit and confirm diagnosis
  Recruit participant(s) based on well‐defined inclusion and exclusion criteria. Prior to trial initiation, confirm clinical diagnosis using standardized diagnostic procedures, ensuring that the participant(s) is (are) eligible and appropriately classified.
Step 6 – adopt randomization, concealment and masking
  Implement randomization, allocation concealment strategies (to minimize selection bias) and masking techniques to reduce performance and detection bias, where feasible.
Step 7 – implement the interventions and collect data
  Implement interventions strictly in accordance with the trial protocol. Standardize data collection methods to ensure consistency and reliability across treatment cycles. Record systematically, with attention to completeness and temporal alignment with intervention periods.
Step 8 – analyse the data
  Apply the previously defined statistical analyses to each participant's data. Tailor analysis methods to detect individual treatment effects and include strategies for interpreting outcomes in relation to the original research question. Consider data visualization and effect size estimation.
Step 9 – write up the manuscript (use CENT)
  The reporting of each trial should adhere to the CENT (CONSORT Extension for N‐of‐1 Trials) guidelines. Draft manuscripts and revise them to ensure clarity, engagement and completeness. Include essential information regarding methodology, results and interpretation, providing a transparent and thorough account of the trial.
Step 10 – publish trial and share/deposit data
  Following manuscript preparation, submit results to peer‐reviewed journals appropriate to the clinical topic. In parallel, deposit de‐identified trial data in publicly accessible repositories to promote transparency, reproducibility and future secondary analysis.

**FIGURE 3:**
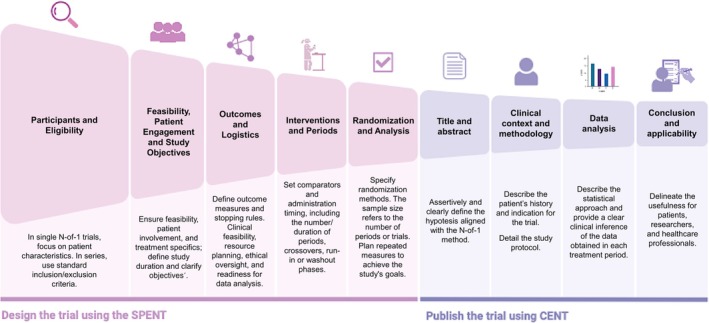
Visual representation of the Main steps in trial design and report, following SPENT and CENT recommendations. SPENT: SPIRIT extension for N‐of‐1 trials reporting guideline and checklist. CENT: CONSORT extension for reporting N‐of‐1 trials.

Figure 3 provides a visual representation of the main steps in trial design, following SPENT guidelines and manuscript preparation according to CENT recommendations. We refer the reader to the original guidelines^16^, ^28^ for an in‐depth understanding of all the mentioned steps. Henceforth, we focus on the statistical analysis of N‐of‐1 trials, a topic we believe has been underexplored in the literature.

## 3 STATISTICAL ANALYSIS

Statistical analysis for evaluating changes in N‐of‐1 trials is constantly developing. N‐of‐1 data have unique features that challenge standard statistics, requiring specific tests. Several statistical tests are suitable, each with advantages and limitations. As the field evolves, the circumstances that are more appropriate for each test become clearer.

Although the randomized design of an N‐of‐1 trial structures the measurements, the time series may exhibit trends and patterns that challenge the interpretation of the results. An existing time trend or a periodic process independent of the intervention may increase or decrease the variable of interest, leading to erroneous conclusions if not accounted for in data analysis.^32^

Another issue is data autocorrelation, which occurs when a previous measurement influences the current measurement and is common in studies that record repeated measures of the same individual. Ignoring an autocorrelation in the opposite direction of the treatment effect can inflate standard errors and hinder the identification of an actual effect (Type II error), whereas ignoring an autocorrelation in the same direction as the treatment effect can underestimate standard errors and lead to the identification of a nonexistent effect (Type I error).^33^ Conventional statistical tests, such as t‐tests, require independent data points. Such an assumption does not hold for autocorrelated data, which explains why commonly used statistical tests do not apply to N‐of‐1 trials.^34^

A carryover effect occurs when treatment effects linger beyond the crossover (i.e., when the subsequent treatment starts). Inserting a washout period (i.e., without treatment) between consecutive treatment periods can reduce or even eliminate the carryover effect. Deciding whether to include washout periods depends on the durability of the treatment effect, as well as practical and ethical considerations. Such a period may be necessary to avoid adverse interactions between the treatment conditions. It is also possible to address carryover effects by eliminating or downweighing observations taken at the beginning of a treatment period.^15^ Focusing on scenarios involving many measurements throughout the study, a Bayesian distributed lag model has been designed to estimate carryover effects while incorporating temporal correlations through an autoregressive model. This approach demonstrated improved accuracy in estimating carryover effects compared to existing methods.^35^

As an N‐of‐1 trial has a participant sample size of one, the statistical power is a function of the number of repeated measurements from that same individual over time. Increasing the number of measures enhances study power but can impose an extra burden on the participant, limiting the power manipulation in N‐of‐1 trials.

### 3.1 Time series data removal and modelling approaches

Various time series analysis techniques address time‐related patterns and autocorrelation, either removing or modelling them during the statistical analysis so that researchers can apply parametric tests to the ‘prewhitened’ data.^36^ Unless used very carefully, transforming data can remove effects of interest, hindering the identification of trends in the data. Cleansing autocorrelation data may distort the graphed data and impact visual analysis. Auto‐Regressive Integrated Moving Average (ARIMA), dynamic regression modelling and a Bayesian framework are examples of modelling approaches.^15^

ARIMA is the best‐established method for controlling autocorrelation,^37^, ^38^ finding solutions iteratively through maximum likelihood methods. It provides the mean or the magnitude of intervention coefficients as an effect size estimator, but requires a long data series. Small‐Sample Forecasting Regression provides more precise regression estimates when n = 20 (20 repeated measures) than the ARIMA estimates when n = 30.^39^ Nevertheless, many N‐of‐1 trials record fewer measures than the Small‐Sample Regression Model requires.

Dynamic regression modelling is an ordinary least squares (OLS) regression model. It accounts for autocorrelation in the data by incorporating dynamic (time‐varying) variables that capture the past's influence on the outcome and predictors in the model. It integrates time trends and periodicity and is appropriate for repeated measurements of at least 50 data points.^40^ Regression coefficients and model fit statistics indicate the intervention's effect size. A shortcoming is that data series with a few repeated measures, typical in single‐case research, do not meet the OLS assumptions of constant variance, normality and linearity of relationship.^41^ A didactic tutorial paper describes a simple 10‐step procedure for analysing N‐of‐1 observational data via dynamic regression modelling in SPSS.^33^

Bayesian models do not rely on asymptotic normality for drawing inferences; they use a prior distribution to combine subjective assessment of treatment efficacy with experimental data to develop a posterior evaluation needed for clinical decision‐making. The posterior means of intervention effects and the Bayesian model comparison metrics estimate the intervention's effect size.^42^ Bayesian modelling of N‐of‐1 data may be a complex statistical procedure for individuals without in‐depth knowledge of Bayesian analysis. Although Bayesian methods can model data with small sample sizes, when the sample size is as small as N = 20, they provide a more biased estimate than a maximum likelihood regression model.^43^ Recent developments include hierarchical Bayesian structures that facilitate adaptive designs, using population estimates to improve individual‐level treatment effect estimation.^44^

### 3.2 Effect size estimators: parametric and nonparametric tests

The several measures for estimating treatment effect sizes from SCED data can be divided into parametric and nonparametric measures. The most prevalent among the parametric measures is the standardized mean difference (SMD), which originates from Cohen's d metric.^45^ The SMD assumes autocorrelation but considers that the data series shows no trend over time. Therefore, it overestimates the variance when there is a significant negative or positive autocorrelation.^46^ The Log Response Ratio (LRR) has recently been developed.^47^ As the natural logarithm of the response ratio, it relies on a simple model where the outcome level is stable within each period for comparing two periods. As autocorrelation in the data series will bias estimates of the sampling variance of the effect size for a single case study, nonparametric options are available if the researcher doubts the validity of the parametric assumption in a particular dataset.

In the literature, nonparametric measures of association have been referred to as effect size estimators, even though they do not always measure the magnitude of an effect.^48^ Nonparametric tests do not assume a specific data distribution, making them more flexible and robust when dealing with small samples or when the data do not meet parametric assumptions. Several effect size measures for SCEDs exist, such as the Nonoverlap of All Pairs (NAP),^49^ Percent of Non‐overlapping Data (PND)^50^ and between‐case Standardized Mean Difference.^51^ NAP derives from receiver operator curve (ROC) analysis as an area under the curve and equals the PND, which has been criticized for being based on only one data point in the baseline phase and, therefore, is overly influenced by outliers.^52^ The Percentage of Data Points Exceeding the Median,^53^ the percent of all non‐overlapping data (PAND),^54^ and the Improvement Rate Difference (IRD)^55^ do not have this shortcoming. Nevertheless, they cannot control for the baseline trend.

A baseline trend is expected in SCED data.^56^ Other measures, such as the Percentage of Data Points Exceeding the Median Trend (PEM‐T)^57^ and Tau‐U,^33^ have been developed to consider trends in the dataset. Both are nonoverlap indices, which reflect the non‐overlapping data between the baseline and intervention. They differ in how they handle (across phases) and combine overlapping *vs*. non‐overlapping data pair counts. PEM‐T considers the baseline trend. It consists of drawing a trend line in the baseline period via a split‐middle technique and extending it to the treatment period.^58^ The non‐overlap effect size estimator is the percentage of data points in the treatment period above the trend line. Tau‐U has become a popular statistical technique for single‐case experimental designs.^59^ It also belongs to the Nonoverlap indices. Nevertheless, they all compare individual data points across periods A and B to evaluate the “dominance” of one score set over the other.^60^

Tau‐U derives from Tau Kendall's Rank Correlation and the Mann–Whitney U test. It allows controlling the undesirable period A (baseline) trend and indicates the percentage of non‐overlapping data minus the percentage of overlapping data^32^ and is well suited for small datasets because it exceeds parametric techniques when the data do not conform with parametric assumptions. Indeed, it is a family of four indices: (a) A *vs*. B period nonoverlap (very similar to the nonoverlap of all pairs (NAP): the percentage of data showing improvement between periods A and B); (b) nonoverlap with the baseline trend controlled (A *vs*. B ‐ Trend A or Tau‐U Trend A), which indicates the overall improvement, controlling for preexisting (baseline or period A) improvement trends in the entire data series; it is the most related to the regression control method^61^ and shows better discriminability and sensitivity than the other nonoverlap measures; c) nonoverlap and period B trend together, which indicates the percentage of data showing improvement between A and B and within period B; (d) nonoverlap and period B trend with the baseline trend controlled (A *vs*. B + Trend B ‐ Trend A), indicating the percentage of data that improve over time considering the period nonoverlap and period B trend, after controlling for the period A trend.

Tau‐U estimates are not bound between −1.00 and 1.00, making them difficult to interpret as effect size estimators.^59^ A second baseline‐corrected Tau‐U^62^ removes the baseline trend with Theil‐Sen robust regression. This alternative measure varies between −1.00 and 1.00, but it fails to control the trend when there are too few measurements (e.g., n = 5) in the baseline period.^59^

### 3.3 Most used statistical methods

A recent systematic review examining publications of medical N‐of‐1 trials indicated that most studies (99.1%) fail to report an appropriate design‐comparable effect size or a confidence/credible interval, ignore autocorrelation (83.8%) and do not meet distributional assumptions (65.8%); 47.9% do not report the raw data, rendering meta‐analysis impossible.^63^

Researchers conducting N‐of‐1 trials seem to prefer straightforward analysis methods. A systematic review of N‐of‐1 trials in the medical field revealed that 52% of the studies used visual analysis, 44% used t tests and 24% used nonparametric methods.^64^ Another review reported that 75% of the reports performed or planned statistical analyses, 53% used paired t tests and 32% used a nonparametric method.^65^

Although conventional t‐tests are not appropriate for time series data, serial t‐tests for level and rate changes that account for serial correlation in a single individual have been recently developed. When serial correlation is positive, finding a “statistical difference” is less likely when using conventional t tests than a method that accounts for serial correlation (e.g., serial t tests).^66^ Serial t‐tests require at least four pairs of treatments, provide effect size and precision calculations and can be incorporated into future studies.

A recent review of randomized N‐of‐1 trials conducted over twelve years identified a wide range of data analysis approaches, including regression models (23.0%), t tests (24.0%), Bayesian approaches (14.9%), nonparametric analyses (12.2%), graphs or visual inspection of data (5.4%), no formal analysis methods (15.4%) or no methods of analysis (11.5%).^67^

Barnard‐Brak et al.^68^ examined the degree of autocorrelation among single‐case design data with the most commonly used effect size measures. While the degree of autocorrelation substantially influenced PND, NAP and IRD, Tau‐U was not significantly influenced by autocorrelation. Parametric measures of effect sizes, such as SMD and LRR, were also not significantly influenced by the degree of autocorrelation.

### 3.4 Aggregating N‐of‐1 studies

As N‐of‐1 research focuses on individual studies, one can even question whether any information from one patient is applicable to another. Nevertheless, it is possible to conduct a series of N‐of‐1 trials and use statistical methods that enable the aggregation of N‐of‐1 studies, allowing for the estimation of both group‐ and individual‐level effects.^33^ Appropriately powered N‐of‐1 trials permit the exploration of the impact of an intervention on more than one outcome (i.e., multivariate N‐of‐1 trials). Formulae for calculating sample sizes and related quantities for sets of N‐of‐1 trials have already been developed.^69^

Traditional meta‐analytical techniques cannot synthesize non‐overlap measures. Researchers can use parametric measures, hierarchical linear modelling and estimate design‐comparable effect sizes for meta‐analysis when it is possible to include enough repeated measures in the study design. A systematic evaluation of approaches for the statistical analysis of a series of N‐of‐1 trials revealed that all the evaluated regression models, Bayesian Networks, G‐estimation and a carryover‐adjusted parametric model (COAPM) perform well when there is no carryover and no treatment dependence.^70^

**FIGURE 4:**
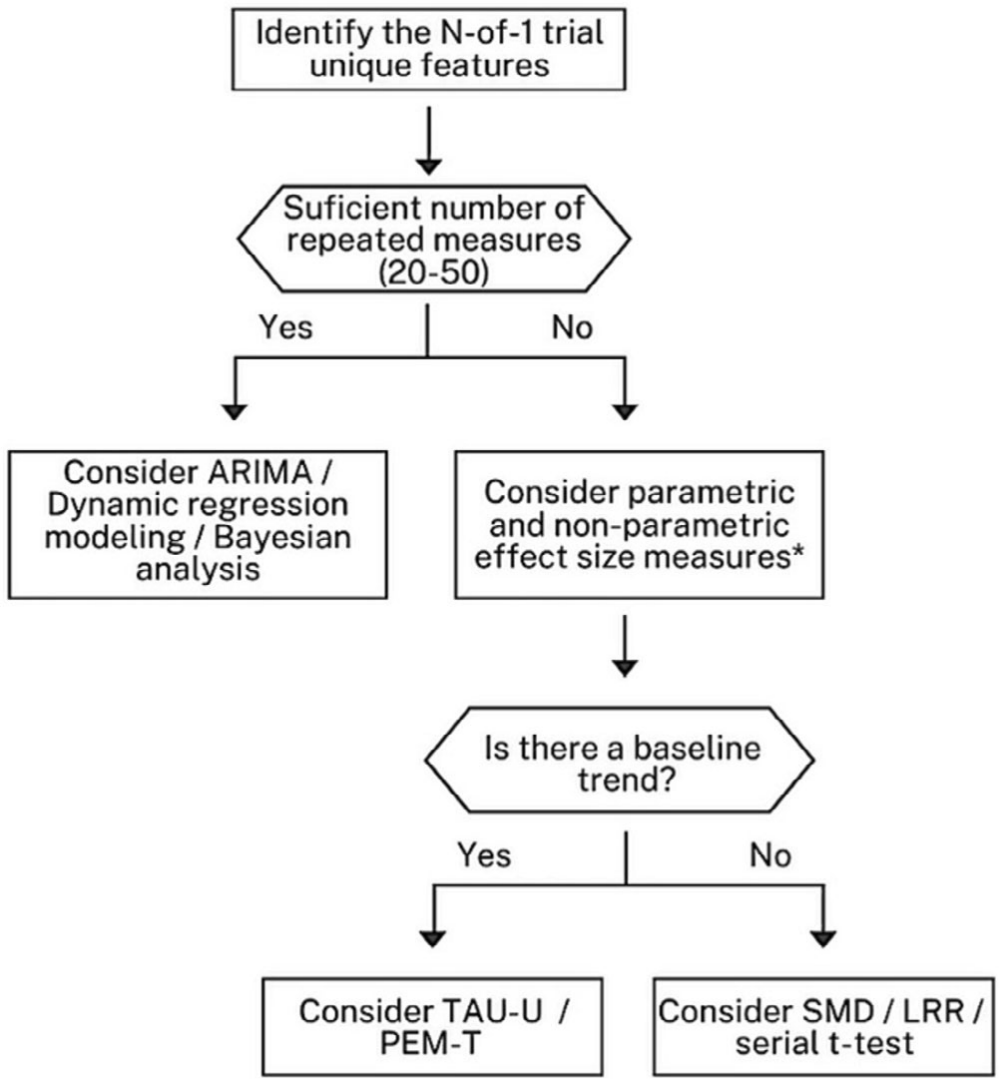
Flowchart of statistical analysis options for N‐of‐1 trials. Visual analyses complement statistical findings, especially in small samples. Ensure decision points reflect the nuances of your study, including ethical considerations regarding washout periods and the burden of data collection. Adapt the flowchart according to the specific study designs and participant characteristics. Document findings thoroughly, as many studies lack proper reporting of effect sizes. *improvement rate difference (IRD), nonoverlap of all pairs (NAP), percent of all non‐overlapping data (PAND), percentage of data points exceeding the median trend (PEM‐T), percent of non‐overlapping data (PND), tau‐U, log response ratio (LRR), standardized mean difference (SMD).

We provide a flowchart summarizing this section to help researchers navigate the complexities of statistical analysis in N‐of‐1 trials (Figure 4). Table 1 summarizes the main statistical measures and modelling approaches.

**TABLE 1:** Summary of the Main statistical measures and modelling approaches for N‐of‐1 trials.

TYPE	MEASURES	DESCRIPTION	REFERENCE
Modelling Approaches	Auto‐Regressive Integrated Moving Average (ARIMA)	Arima is a time series model to analyse autocorrelated data.	Box et al., 2015^39^
	Bayesian Modelling	It uses prior distributions to estimate treatment effects.	Schmid & Yang, 2022^42^
	Dynamic Regression Modelling	Regression Modelling incorporates time‐varying variables to analyse data trends and autocorrelation.	Keele & Kelly, 2006^40^
Nonparametric, Nonoverlap Measures	Improvement Rate Difference (IRD)	IRD measures the difference in the rate of improvement between treatment periods.	Parker et al., 2009^55^
	Nonoverlap of All Pairs (NAP)	NAP compares pairs of data points across treatment periods to assess improvement.	Parker & Vannest, 2009^49^
	Percent of All Non‐overlapping Data (PAND)	PAND is a derivative of PND that includes all data points to assess nonoverlap between treatment periods.	Parker et al., 2007^54^
	Percentage of Data Points Exceeding the Median Trend (PEM‐T)	PEM‐T consists of drawing a trend line in the baseline period using a split‐middle technique and extending it to the treatment period	White & Haring, 1976^57^
	Percent of Non‐overlapping Data (PND)	PND measures the percentage of data points in the treatment period that do not overlap with baseline data.	Scruggs et al., 1987^50^
	Tau‐U	Tau‐U accounts for baseline trends while assessing nonoverlap.	Parker et al., 2011^32^
Parametric Measures	Log Response Ratio (LRR)	LRR compares the logarithm of response ratios to estimate effect sizes.	Pustejovsky et al., 2014^51^
	Serial t‐test	Serial t‐test accounts for serial correlation in a single individual.	Tang & Landes, 2020^66^
	Standardized Mean Difference (SMD)	SMD measures the size of the difference between two treatment periods in standard deviation units.	Busk & Serlin, 1992^45^

### 3.5 Implementation challenges

N‐of‐1 trials face several practical and theoretical challenges that limit their widespread use in generating medical evidence and in clinical practice. One of the main barriers is the complexity of their design, which requires careful planning, multiple treatment cycles and frequent data collection. This process demands considerable time and effort from both clinicians and patients, which may be difficult to manage in routine care settings.^71^ As a result, successful N‐of‐1 trials depend on highly motivated, proactive and reliable patients, as well as clinicians who are familiar with the methodology and willing to invest the necessary effort. However, both groups often lack familiarity with the N‐of‐1 concept, and there may be resistance to adopting what is perceived as a research‐oriented approach in routine care.^72^ Additionally, ensuring high‐quality, consistent data collection over multiple periods is challenging, especially when relying on patient‐reported outcomes.^73^ Finally, N‐of‐1 trials often require a more active role from the patient in treatment decisions, which may shift the traditional dynamic of the patient‐physician relationship.^71^

Infrastructure is another critical element for the successful implementation of N‐of‐1 trials, as it must meet the requirements of random allocation, blinding, data collection, monitoring and related processes.^3^ However, most clinical settings lack dedicated platforms or workflows tailored to the demands of N‐of‐1 design, without which these trials remain difficult to scale or integrate into routine care.^5^ Moreover, the absence of standardized protocols and centralized platforms limits replication, quality control and multi‐site collaboration.^15^ Addressing these gaps in infrastructure is essential to move from isolated academic examples to broader clinical adoption.^74^

While practical and infrastructural barriers are significant, theoretical challenges also contribute significantly to limiting the broader adoption of N‐of‐1 trials. Ensuring methodological rigour is particularly complex, as issues like carryover effects, period effects and the selection of appropriate outcome measures can complicate interpretation. Determining optimal allocation sequences, blinding strategies and washout periods is often difficult—especially for interventions with long‐lasting or delayed effects.^11^

Moreover, while statistical tools for N‐of‐1 analysis have evolved considerably, their proper application remains a barrier in practice. Most N‐of‐1 trials fail to report appropriate design‐comparable effect sizes or confidence/credible intervals, and many do not account for autocorrelation or meet distributional assumptions. These deficiencies can result in potentially erroneous effect size estimations.^63^ These issues are discussed in more detail in the statistical analysis section of this article.

Additionally, translating individualized findings into broader clinical recommendations remains difficult. Although statistical methods for aggregating N‐of‐1 data exist, they are not yet standardized, and their use in informing guidelines or regulatory decisions remains limited.^1^

Ethical and regulatory questions further complicate implementation. Because these trials exist at the intersection of clinical care and research, they raise important concerns about clinical equipoise, informed consent, reporting and funding.^75^ Obtaining IRB approval can also be time‐consuming, particularly when the trial involves novel interventions or data collection methods.^3^ These barriers demonstrate the complexity of integrating N‐of‐1 trials into routine care, while also identifying key areas for practical innovation.

### 3.6 Future perspectives

Future perspectives for N‐of‐1 trials include their role in precision medicine and the refinement of evidence‐based practices. Achieving the full potential of N‐of‐1 trials requires the collaborative development of universally accepted processes, platforms, methods and standards that seamlessly integrate into the current healthcare ecosystem. This effort calls for cooperation among stakeholders, including clinicians, patients, researchers and healthcare payers, to establish sustainable value‐based arrangements that promote the widespread adoption of N‐of‐1 trials.^3^ Through the progression of digital health technologies, there is potential for these trials to be conducted on a larger scale and with greater ease. Digital tools and platforms can facilitate the design and conduct of N‐of‐1 trials, providing innovative ways to assess individual treatment effects and personalize treatment strategies, making them more accessible to clinicians and researchers.^76^, ^77^ Additionally, addressing methodological issues such as the appropriate use of statistical methods and reporting standards is crucial for the accurate interpretation of trial results and for facilitating meta‐analyses.^63^

The growing use of N‐of‐1 designs in precision and personalized medicine reflects a shift toward tailoring interventions to the specific needs of individuals, especially in areas with substantial patient heterogeneity. This is particularly evident in the development of genetic N‐of‐1 therapies, which have shown promise for rare diseases caused by unique variants. These interventions challenge conventional regulatory paradigms and require new ethical frameworks and pragmatic pathways for clinical integration.^78^ Advances in this area include comprehensive baseline characterization, individualized outcome assessments and robust statistical analysis.^1^ There are also applications emerging in fields such as neuro‐oncology^79^ and personalized nutrition.^80^

A key enabler of this expansion is the democratization of N‐of‐1 trials through digital platforms, which can streamline trial design, data collection and analysis. Platforms like StudyU and StudyMe demonstrate the feasibility of integrating user‐friendly, secure and scalable digital environments into routine practice, increasing patient engagement and accelerating research dissemination.^76^, ^77^ New statistical approaches, including adaptive designs, Bayesian approaches and sequential monitoring, are improving the efficiency and data quality of N‐of‐1 trials. These innovations allow for early stopping rules, improved power and aggregation of data across multiple N‐of‐1 trials to inform both individual and population‐level treatment decisions.^81^

Additionally, the integration of multi‐omics data into N‐of‐1 designs enables deep phenotyping and the mechanistic understanding of treatment responses. As highlighted in a recent review, the convergence of omics technologies, artificial intelligence and secure data platforms—such as blockchain—can transform individualized research by enabling richer, longitudinal data collection and analysis. These tools may enhance the capacity of N‐of‐1 trials to model complex biological systems, refine therapeutic strategies and support the evolution of omics‐informed precision care.^82^ These developments point to a promising future for N‐of‐1 trials in personalized medicine and support their broader acceptance by clinicians and researchers.

## 4 CONCLUSION

N‐of‐1 trials offer a rigorous framework for evaluating individual treatment responses and hold considerable promise for advancing personalized medicine. This article has outlined their methodological foundations, statistical approaches and the key challenges to implementation in clinical practice. Despite their potential, wider adoption is hindered by practical, infrastructural and theoretical barriers. Addressing these will require methodological standardization, digital infrastructure and greater integration into routine care. With continued innovation and collaboration, N‐of‐1 trials can play a pivotal role in reshaping evidence generation and tailoring treatments to individual patients.

## AUTHOR CONTRIBUTIONS

MCLM and LLOS conceived and organized the review, analysed publications and helped draft the manuscript. UCA contributed to the conceptualization of the review and revised the manuscript critically and in detail. ABVJ was responsible for reviewing the statistical analysis section. CNSS and MMDS contributed to the overall review and refinement of the manuscript. All authors read and approved the final version of the manuscript.

## CONFLICT OF INTEREST STATEMENT

The authors declare no conflict of interests.

## Data Availability

Data sharing not applicable to this article as no datasets were generated or analysed during the current study.
